# Investigation of dissimilar metal welds by energy-resolved neutron imaging

**DOI:** 10.1107/S1600576716006725

**Published:** 2016-06-09

**Authors:** Anton S. Tremsin, Supriyo Ganguly, Sonia M. Meco, Goncalo R. Pardal, Takenao Shinohara, W. Bruce Feller

**Affiliations:** a Space Sciences Laboratory, University of California at Berkeley, 7 Gauss Way, Berkeley, CA 94720, USA; b Cranfield University, Cranfield, Bedfordshire MK43 0AL, England; c Japan Atomic Energy Agency, 2-4 Shirakata-shirane Tokai-mura, Naka-gun, Ibaraki, 319-1195, Japan; d NOVA Scientific Inc., 10 Picker Road, Sturbridge, MA 01566, USA

**Keywords:** nondestructive testing, laser welding, dissimilar joining, microstructure, neutron imaging

## Abstract

A nondestructive study of the internal structure and compositional gradient of dissimilar metal-alloy welds through energy-resolved neutron imaging is described in this paper. The ability of neutrons to penetrate thick metal objects (up to several cm) provides a unique possibility to examine samples which are opaque to other conventional techniques. The presence of Bragg edges in the measured neutron transmission spectra can be used to characterize the internal residual strain within the samples and some microstructural features, *e.g.* texture within the grains, while neutron resonance absorption provides the possibility to map the degree of uniformity in mixing of the participating alloys and intermetallic formation within the welds. In addition, voids and other defects can be revealed by the variation of neutron attenuation across the samples. This paper demonstrates the potential of neutron energy-resolved imaging to measure all these characteristics simultaneously in a single experiment with sub-mm spatial resolution. Two dissimilar alloy welds are used in this study: Al autogenously laser welded to steel, and Ti gas metal arc welded (GMAW) to stainless steel using Cu as a filler alloy. The cold metal transfer variant of the GMAW process was used in joining the Ti to the stainless steel in order to minimize the heat input. The distributions of the lattice parameter and texture variation in these welds as well as the presence of voids and defects in the melt region are mapped across the welds. The depth of the thermal front in the Al–steel weld is clearly resolved and could be used to optimize the welding process. A highly textured structure is revealed in the Ti to stainless steel joint where copper was used as a filler wire. The limited diffusion of Ti into the weld region is also verified by the resonance absorption.

## Abstract 2

Energy-resolved neutron imaging is used for a nondestructive study of bulk internal microstructure, elemental composition and distribution of voids in dissimilar metal-alloy welds of ∼10 mm thickness. All these characteristics are measured simultaneously in one experiment with a few hundred micrometre spatial resolution.

## 1. Introduction   

The ability of neutrons to penetrate relatively thick metal objects allows the use of various nondestructive testing techniques where other more conventional methods (*e.g.* laboratory X-rays) cannot be implemented. Neutron imaging with thermal and cold neutrons provides unique contrast and is often used to study internal structure and the presence of defects in various metal samples (Kockelmann *et al.*, 2007 ▸; Bilheux *et al.*, 2009 ▸; Kardjilov *et al.*, 2012 ▸; Lehmann *et al.*, 2009 ▸), as well as the distribution of hydrogen-containing substances within metals (Grosse *et al.*, 2013 ▸; Griesche *et al.*, 2015 ▸; Tremsin, Morgano *et al.*, 2015 ▸), water in proton exchange membrane fuel cells (Fu *et al.*, 2011 ▸; Stahl *et al.*, 2015 ▸), coolant in heat pipes (Cimbala *et al.*, 2004 ▸), diesel in fuel injectors (Lehmann *et al.*, 2015 ▸) and many others. At the same time, neutron diffraction is widely used for studies of crystallographic properties as the wavelengths of thermal and cold neutrons are comparable to lattice parameters (several ångströms) (Vogel, 2000 ▸; Santisteban *et al.*, 2001 ▸, 2002 ▸; Hutchings *et al.*, 2005 ▸). Measurements of strain, texture and mosaicity and the identification of various metallographic phases and defects within samples opaque to other particles and X-rays are often conducted with neutrons at the facilities dedicated to strain and texture scanning at both continuous and pulsed neutron sources. At the same time, the elemental composition of various samples can be studied through the resonance absorption, which typically appears in the epithermal range of energies (Postma & Schillebeeckx, 2005 ▸; Schillebeeckx *et al.*, 2012 ▸; Kai *et al.*, 2013 ▸, Tremsin *et al.*, 2014 ▸; Festa *et al.*, 2015 ▸). Various nuclei exhibit a specific set of very sharp resonance absorption features in the eV to tens of MeV range of energies, which are available for experimental studies at various spallation neutron sources. The possibility of measuring neutron transmission spectra in a very wide range of energies (spanning from meV to tens of keV) enables simultaneous studies of both crystallographic properties and elemental composition of various metal samples. Providing the transmission spectra can be measured simultaneously in multiple areas of the sample, the variation of these properties across the sample can be investigated in a single experiment.

The recent development of high-resolution neutron counting detectors capable of simultaneous measurement of >250 000 spectra with a spatial resolution of <100 µm (Tremsin, 2012 ▸; Tremsin, Vallerga *et al.*, 2015 ▸) in combination with the present state-of-the-art beamlines at pulsed neutron sources (Mason *et al.*, 2006 ▸; Maekawa *et al.*, 2009 ▸; Mocko *et al.*, 2011 ▸; Kockelmann *et al.*, 2015 ▸) provide the possibility to map both crystallographic properties and elemental composition in various samples with relatively high spatial resolution.

In this paper we demonstrate the possibilities of energy-resolved neutron imaging to study some microstructural features and the bulk distribution of elemental composition in two different sets of dissimilar alloy welds: two autogenous laser overlap welds of Al to steel with different welding parameters, and a Ti to stainless steel butt weld with Cu serving as a filler alloy produced with cold metal transfer (CMT), a state-of-the-art variant of the gas metal arc welding process. The distribution of residual strain in these welds as well as some visualization of texture-related features and the distribution of defects in the welding region are imaged in our experiments with sub-mm spatial resolution. We also demonstrate the possibility of mapping the distribution of various elements within the welds through resonance absorption imaging with epithermal neutrons.

## 2. Experiments   

### 2.1. Measurement of neutron transmission spectra   

The measurements presented in this paper were performed at the Materials and Life Science Experimental Facility (MLF) of the Japan Proton Accelerator Complex (J-PARC) with a pulse repetition rate of 25 Hz. The samples and the detector were installed at the NOBORU beamline (Maekawa *et al.*, 2009 ▸) at a distance of ∼14 m from the source. A collimated neutron beam with ∼0.3° divergence and a wide range of neutron energies was generated by the pulsed source. A high-resolution neutron counting detector (Tremsin, 2012 ▸; Tremsin, Vallerga *et al.*, 2015 ▸) providing both spatial (with a pixel resolution of 55 µm) and temporal (∼80 ns for epithermal and ∼0.5 µs for thermal and cold energies) information from each detected neutron was used in these experiments. The energy of each detected neutron was calculated from its time of flight, *i.e.* the time interval between the generation of the neutron in the spallation process and its arrival at the detector plane. The short duration of the neutron pulses at the MLF facility (two pulses of 100 ns width separated by 600 ns; Hasemi *et al.*, 2015 ▸; Tremsin *et al.*, 2014 ▸) and the high brightness of the neutron beam were also very important for the present experiments, where neutron transmission spectra were measured in each of the 55 × 55 µm pixels of the detector, 262 144 of them simultaneously. The latter enabled spatial mapping of crystallographic properties within the welds through the analysis of Bragg scattering within the samples. At the same time, the elemental composition and intermetallic compound formation in the weld region were analysed with a relatively high spatial resolution through neutron resonance absorption. The detector used in the present experiments has an active area of 28 × 28 mm and is capable of simultaneous detection of multiple (tens of thousands of) neutrons, as the 512 × 512 pixels in the imaging device function independently of each other. The latter is crucial for the detection of neutrons in the epithermal range of energies, where multiple particles arrive at the detector in the first ∼100 µs after the spallation. No chopper was present in the beam, and the high flux of fast neutrons and gamma photons at the time of spallation was filtered by a 5 cm bismuth filter, installed upstream in the beam, approximately 4 m from the detector. The high detection efficiency of our device at thermal (∼50%) and cold (∼70%) energies (Tremsin *et al.*, 2011 ▸) was also very important for the experiments where limited neutron flux required substantial integration time in order to acquire sufficient statistics for measured neutron spectra in each small area of the sample. However, the efficiency of the detector in the epithermal range is relatively low (<1%) and is expected to be improved by a collaboration between Nova Scientific Inc. and the University of California at Berkeley. The detector used in the experiment contained a 2 × 2 array of Timepix readout (Llopart *et al.*, 2007 ▸) and a stack of neutron-sensitive microchannel plates (MCPs). The MCPs converted each incoming neutron into an electron avalanche consisting of ∼10^5^ electrons, all contained within a ∼10 µm diameter pore, thus providing intrinsic event localization within that pore. The fast readout electronics with 320 µs readout time enabled acquisition of multiple shutters for each neutron pulse with individually controlled time resolution within each shutter (Tremsin, 2012 ▸; Tremsin, Vallerga *et al.*, 2015 ▸), allowing optimization of the detector operation for epithermal, thermal and cold ranges of energies, acquired all in one measurement.

The weld samples were installed ∼15 mm from the detector active area (∼3 mm from the detector vacuum enclosure). This relatively small distance between the samples and the detector active area allowed imaging with high spatial resolution as degradation of the image due to the ∼0.3° beam divergence was rather limited. All measured transmission spectra were normalized by the intensity of the incoming neutron beam, which was measured with no samples installed in front of the detector (often referred to as ‘open beam normalization’). A substantial spatial variation of measured transmission spectra for the ‘open’ beam caused by the high degree of mosaicity of the bismuth filter and the energy dependence of the incoming neutron flux were thus eliminated by this normalization procedure.

### 2.2. Dissimilar alloy weld samples   

#### 2.2.1. Aluminium to steel joint   

**Figure 1:**
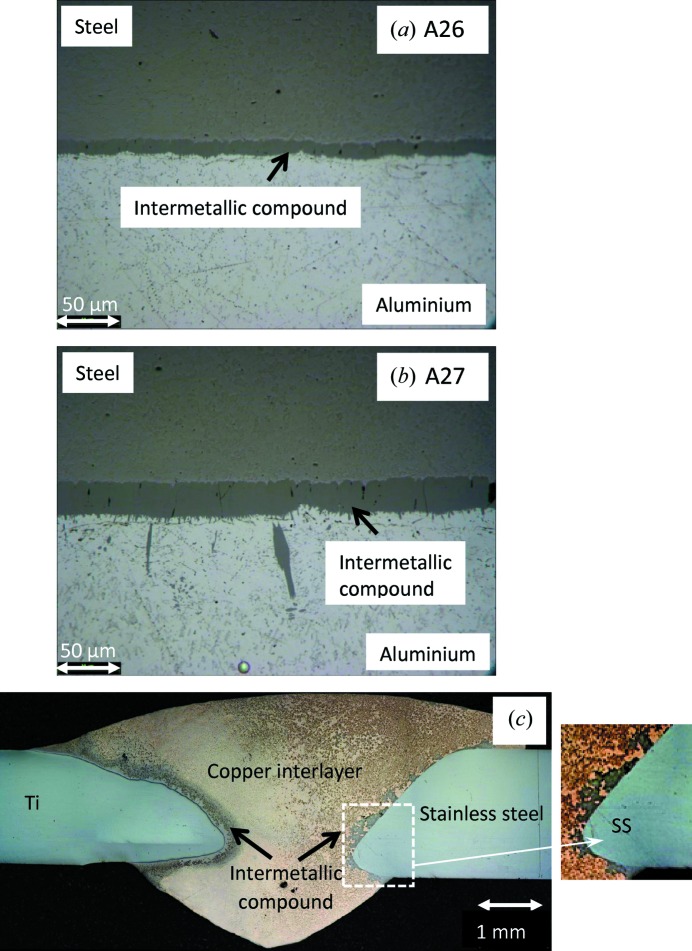
(*a*), (*b*) Micrographic images of steel to aluminium welds (samples A26 and A27) showing intermetallic compound formation near the interface. (*c*) Micrographic image of titanium to stainless steel joint with copper as an interlayer.

The Al–steel joints were welded in an overlap configuration with steel positioned on the top. The principle followed in joining was to create a suitable thermal gradient by which the heat would be conducted from the steel to the aluminium surface and the lower-melting-point aluminium would only be molten in the interface region and wet the steel substrate. By ensuring that the steel near the interface does not melt, we can prevent aggressive inter-diffusion of Fe and Al atoms resulting in the formation of intermetallic compounds, *e.g.* FeAl_3_, Fe_2_Al_5_. The plates were 150 mm long and 138 mm wide. The steel was 2 mm thick and the aluminium was 6 mm thick. A continuous-wave Yb fibre laser (IPG Photonics) with 8000 W of maximum power was used with a defocused laser beam of 14.9 mm diameter at the surface of the steel. No shielding gas was used during the joining process. The welding parameters are shown in Table 1 ▸. The details of the welding process and intermetallic formation are given by Meco *et al.* (2015 ▸). Figs. 1 ▸(*a*) and 1 ▸(*b*) show optical micrographs of the cross section, which demonstrate the ∼10–50 µm intermetallic formation along the interface of the dissimilar welded specimens.

#### 2.2.2. Titanium to stainless steel joint   

A titanium AMS4911L plate of dimensions 150 × 100 × 1.7 mm (length × width × thickness) was joined with 316L stainless steel of identical length and width and 2 mm thickness. The plates were joined with a 1 mm diameter CuSi_3_ welding wire deposited using the CMT welding process. The processing parameters are shown in Table 2 ▸. Besides the local shielding provided by the CMT welding torch, a trailing shield with argon was also used to prevent oxidation of the parent alloys, mainly titanium. Ti remains susceptible to oxidation until about 573 K during the cooling cycle, when it comes into contact with atmospheric gases. Fig. 1 ▸(*c*) shows a micrograph of the cross section of this weld. The compositions of the participating alloys for both combinations are shown in Table 3 ▸.

**Figure 2:**
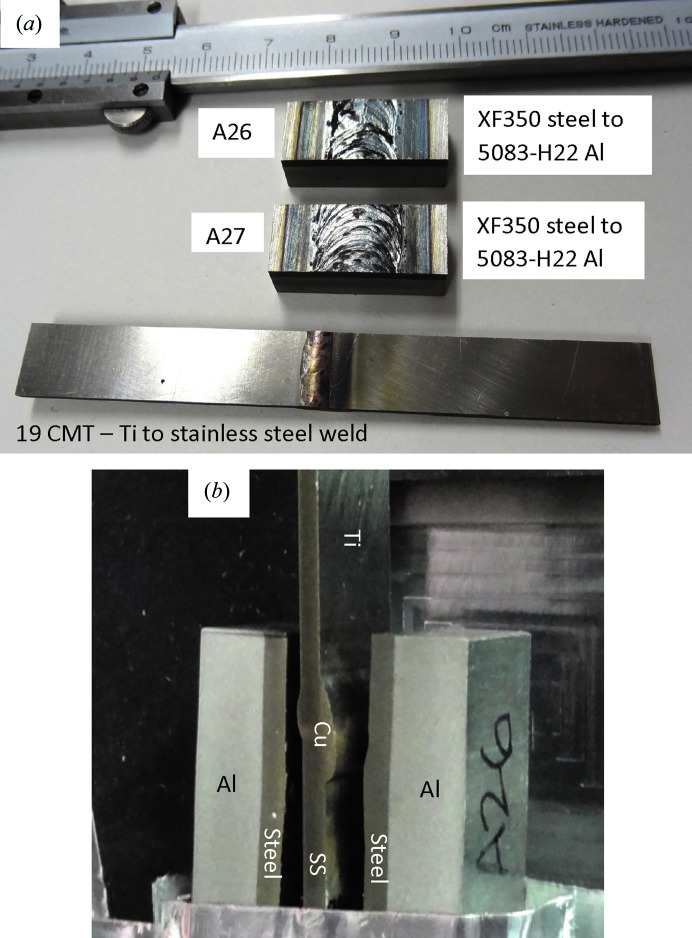
Photographs of dissimilar alloy weld samples used in this study. (*a*) Weld between Ti and stainless steel plates with copper filler (bottom sample) and two steel to aluminium weld samples (steel parts of the weld are visible). (*b*) All three weld samples installed in front of the neutron counting detector in the edge-on orientation.

Fig. 2 ▸ shows photographs of the three welds studied: two Al–steel weld samples, and a Ti–steel weld with copper filler. All three weld samples had 10 mm longitudinal length along the weld direction. The samples were measured in two orientations: with neutrons propagating along the weld direction [as shown in Fig. 2 ▸(*b*)] and in ‘face-on’ orientation, where the neutrons were incident on the sample surface perpendicular to the weld direction, *i.e.* in the orientation facing the beam as shown in Fig. 2 ▸(*a*).

## 3. Discussion   

**Figure 3:**
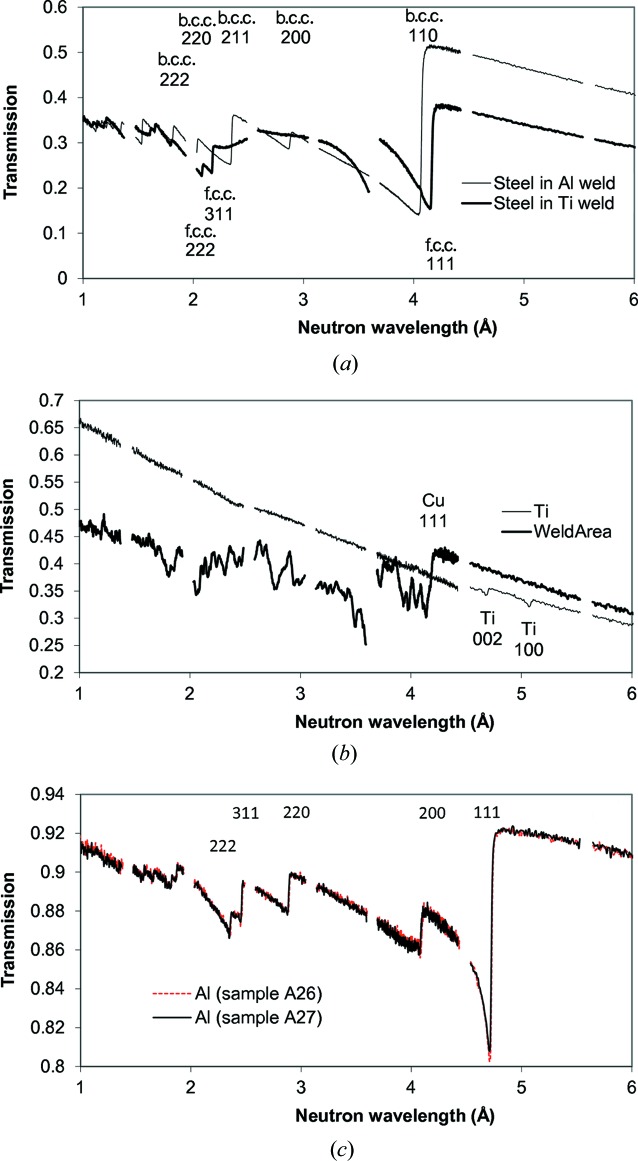
Transmission spectra of weld samples measured at the thermal neutron energies. (*a*) Steel in both welds, (*b*) Ti and the weld region (copper filler), (*c*) Al in two Al–steel welds. The lattice plane indexes (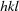) for certain Bragg edges are marked on the graphs. These Bragg edges are used to quantify the residual strain distribution within the samples. The sudden drop in intensity in the Ti to stainless steel weld area seen in spectrum (*b*) indicates the substantial presence of large grains in that area of the weld. No strong diffraction features were observed for the Ti area.

Transmission spectra measured at different areas of the welds are shown in Fig. 3 ▸. The sharp variation of transmission spectra seen in these figures is due to neutron diffraction typical for a polycrystalline material [as in Fig. 3 ▸(*a*)] or for materials with large grains [Fig. 3 ▸(*b*), weld area]. For polycrystalline materials at a certain neutron wavelength the diffraction for a given {*hkl*} plane reaches the maximum angle of 180° and no diffraction happens past that wavelength, leading to a sharp increase of sample transmission. The spectrum of face-centred cubic (f.c.c.) steel in the Ti–steel weld of Fig. 3 ▸(*a*) confirms that there is substantial texture in the steel, which is most likely produced during the rolling process in the manufacture of the steel plate. The spectrum of the copper weld region in Fig. 3 ▸(*b*) clearly indicates that the weld area contains a large number of small grains, which were formed during the welding process. These grains diffract neutrons at certain energies, producing a number of distinct dips in the transmission spectrum. More detailed information on texture can obviously be obtained at a texture-dedicated beamline, such as HIPPO (Matthies *et al.*, 2005 ▸), but the present study reveals the spatial distribution of texture variation with ∼100 µm resolution, over the entire sample in one measurement. At the same time, Fig. 3 ▸(*b*) shows that Ti Bragg edges are quite small and it is very difficult to get quantified information on the microstructure of Ti for such a thin sample.

### 3.1. Energy-resolved neutron transmission radiography of weld samples   

**Figure 4:**
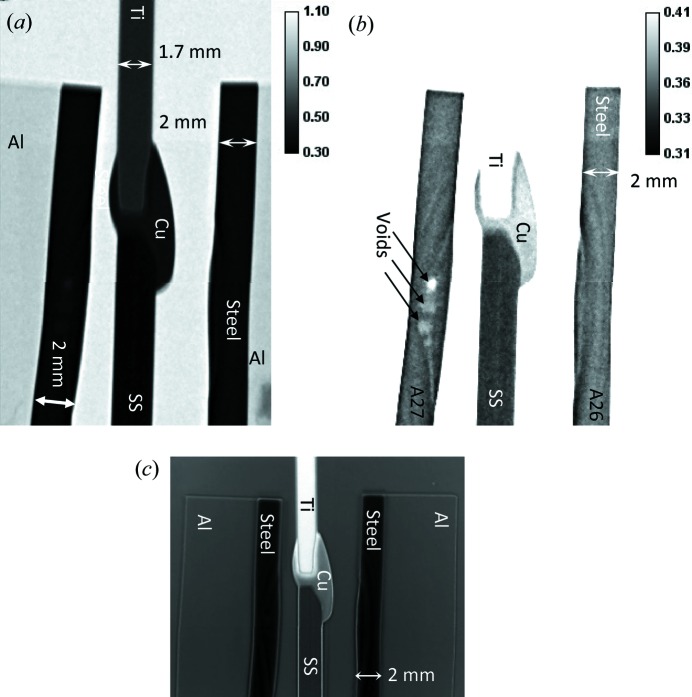
Neutron transmission images of weld samples. (*a*), (*b*) Images are integrated over the entire neutron spectrum of the NOBORU beamline. The two images are identical except for the increased brightness for image (*b*). (*c*) Neutron transmission image of weld samples integrated over the neutron wavelengths 1–4.16 Å normalized by the image obtained past the last Bragg edge (4.18–8 Å). Note the different scale of figure (*c*), where the entire area of weld samples A26 and A27 is shown. A relatively strong refraction at the edges of the samples is seen as bright lines around stainless steel and dark lines around titanium, surrounded by a bright halo, all attributed to refraction. Ti has a negative index of refraction, while for stainless steel it is positive.

**Figure 5:**
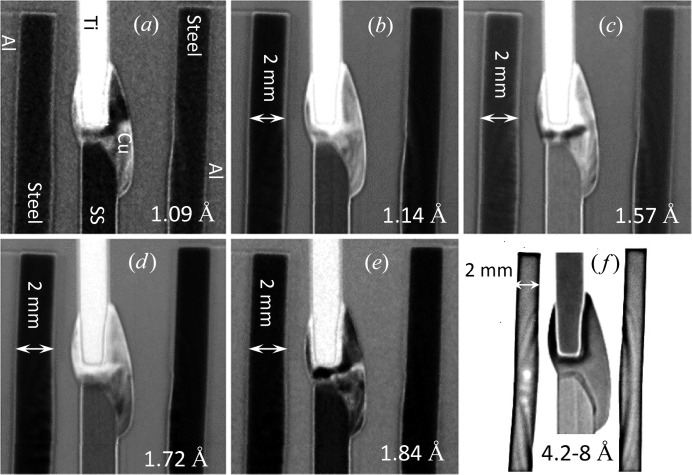
(*a*)–(*e*) Narrow-energy neutron transmission images of weld samples. Only neutrons in a narrow range of energy are used to form these images: (*a*) 1.09 ± 0.003 Å, (*b*) 1.14 ± 0.03 Å, (*c*) 1.57 ± 0.03 Å, (*d*) 1.72 ± 0.18 Å, (*e*) 1.84 ± 0.024 Å. Each of these images is normalized by the neutron transmission image (*f*) acquired past all Bragg edges (4.2–8 Å neutrons), where no diffraction is present in either of the steels or in copper. Image (*f*), shown not at the same scale, reveals the extent of the heat-affected zone within the steel plate. Strong texture is seen in the weld area with copper filler, where dark areas reveal the presence of preferred orientation leading to enhanced diffraction in that area. Some refraction at the Ti part of the weld is seen as a dark line around it. An animation representing a continuous scan through the narrow-band neutron images revealing the texture in the copper weld area can be found in the supporting information online (ks5507sup1.avi).

**Figure 6:**
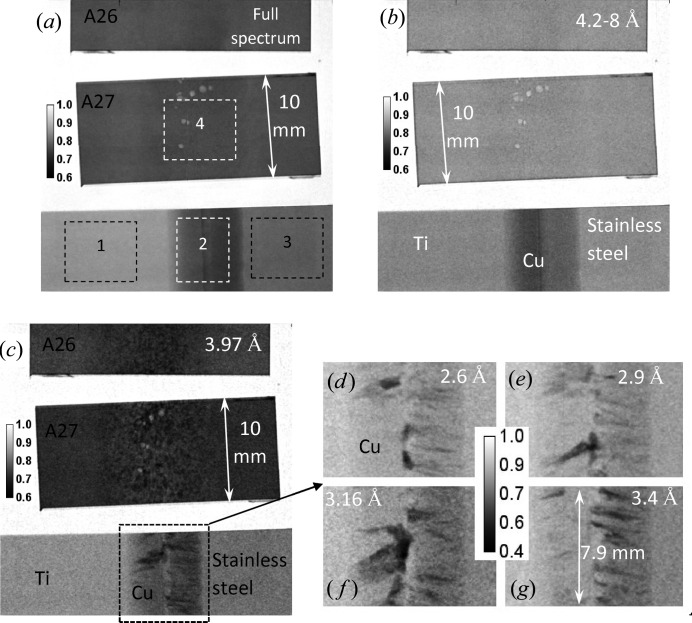
Neutron transmission images of the welds positioned in ‘face-on’ orientation relative to the incoming neutron beam. (*a*) Image integrated over the entire beam spectrum. (*b*) Only neutrons with wavelengths of 4.2–8 Å are used in this image. (*c*)–(*g*) Narrow-energy neutron transmission images of the welds positioned in ‘face-on’ orientation. Image obtained at (*c*) 3.97 ± 0.1 Å, (*d*) 2.615 ± 0.015 Å, (*e*) 2.93 ± 0.03 Å, (*f*) 3.16 ± 0.01 Å, (*g*) 3.435 ± 0.035 Å. The defects seen in Al–steel weld images (*a*) and (*b*) are not due to variation in crystallographic structure of the weld as they are still present in the image taken at neutron energies where no diffraction is present. The texture in the copper weld area with striations perpendicular to the weld line is clearly visible owing to preferred orientation of filler material. Some texture in the melted steel of the Al–steel weld is also observed in image (*c*). The dashed rectangles indicate the areas for which resonance absorption spectra are shown in Fig. 13 ▸.

The white-beam transmission images (images integrated over the entire beam spectrum) shown in Fig. 4 ▸ demonstrate the difference in the integrated neutron transmission of Ti and steel as well as the weld area with copper filler. Fig. 4 ▸(*b*) clearly reveals the presence of voids (indicated by arrows) in the molten area of steel in the Al–steel weld sample shown on the left, where a larger amount of energy was applied during welding. The profile of the heat-affected zone is also visible in the steel part of the Al–steel welds. No microstructure within the copper filler is visible in these images. The images in Figs. 5 ▸(*a*) to 5 ▸(*e*) show the ratio of neutron transmission acquired at the wavelengths where the neutron diffraction takes place, divided by the image acquired at the energies past the last Bragg edge of steel. This normalization enhances the image features formed by the refraction effects. The boundary between the Ti section and copper is visible as a dark line around Ti, which has a negative refraction coefficient. That relatively sharp line confirms the absence of any considerable diffusion of Ti into the copper weld area; otherwise the refraction on Ti would form a white line farther into the Cu filler area and the line itself would not be as sharp as seen in Fig. 5 ▸(*f*). The presence of preferred orientations or grains within the copper filler area is clearly visible in the narrow-energy images of Fig. 5 ▸. The ‘face-on’ images of the welds shown in Fig. 6 ▸ reveal the spatial distribution of needle-like structures within the copper filler as well as the presence of considerable distortions in the steel matrix of the Al–steel sample welded at a higher power [sample A27 in Fig. 6 ▸(*a*)]. The distribution of these features can be revealed with relatively high spatial resolution by the energy-resolved neutron imaging, providing complementary information to the other, conventional, nondestructive testing techniques.

### 3.2. Residual strain mapping through Bragg edge transmission imaging   

**Figure 7:**
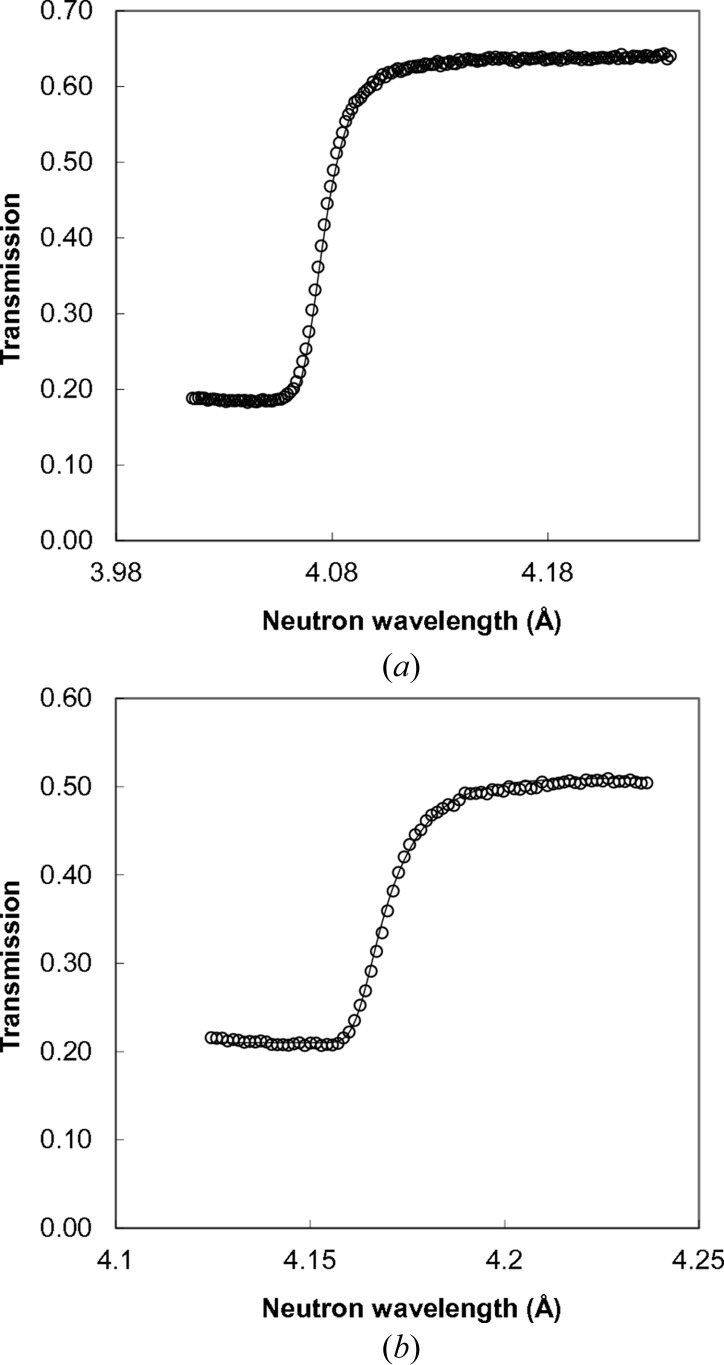
Bragg edges of steel which were used for the measurement of residual strain distribution within steel parts of the welds. (*a*) (110) edge of body-centred cubic (b.c.c.) steel in the Al–steel weld; (*b*) (111) edge of f.c.c. steel in the Ti–stainless steel weld. Markers are the experimentally measured values; the solid line is the analytical function calculated from the equation described by Vogel (2000 ▸), Santisteban *et al.* (2001 ▸) and Tremsin *et al.* (2011 ▸, 2012 ▸).

The sharp variation of neutron transmission, referred to often as Bragg edges, that is caused by neutron diffraction in polycrystalline materials has been used previously to obtain the residual strain distribution across various metal samples (Vogel, 2000 ▸; Steuwer *et al.*, 2001 ▸; Santisteban *et al.*, 2002 ▸; Tremsin *et al.*, 2010 ▸, 2012 ▸). The deficiency of this technique compared to conventional neutron diffraction is the fact that the measured strain represents an integral value through the entire sample in the direction of neutron propagation. The weld samples, however, are among the few cases where the strain distribution integrated along one axis (in particular along the weld seam) can still be useful. The strain distribution with sub-mm spatial resolution across the entire sample can be measured in one experiment. The accuracy of strain reconstruction depends on the energy resolution of the experimental setup and on the number of neutrons acquired in each energy bin. In the present experiment the energy resolution was determined by the width of the initial neutron pulse and the type of moderator used in the beamline (50–90 µs in the thermal range of energies, where the Bragg edges are strongest). This corresponds to an intrinsic energy resolution of ∼0.5% or ∼5000 microstrain. However, fitting an analytical function to the measured Bragg edges can improve the resolution to ∼100 microstrain values, as implemented in earlier analyses (Vogel, 2000 ▸; Steuwer *et al.*, 2001 ▸; Santisteban *et al.*, 2002 ▸; Tremsin *et al.*, 2010 ▸, 2012 ▸). The examples of two edges used in our analysis of residual strain are shown in Fig. 7 ▸. Only one edge of the measured spectra was used in the present analysis, similar to the analysis reported by Vogel (2000 ▸), Steuwer *et al.* (2001 ▸), Santisteban *et al.* (2002 ▸) and Tremsin *et al.* (2010 ▸, 2012 ▸). In principle, a full Rietveld type analysis can be implemented for the reconstruction of microstructure from the measured transmission data (*e.g.* Sato *et al.*, 2011 ▸). However, the challenge of fitting hundreds of thousands of spectra to reconstruct the microstructure distribution within each pixel of our measured data set is yet to be addressed. Existing data analysis tools do not allow such fitting to be finished in a reasonable time. Therefore, in the present study the fitting of a five-parameter analytical function to the measured Bragg edge spectrum around each pixel of our data was implemented with nonlinear least-squares fitting (Tremsin *et al.*, 2010 ▸, 2012 ▸). The spatial resolution of strain maps obtained by the present analysis is determined by both detector spatial resolution and the number of neutrons registered around each Bragg edge used in the fitting. In theory, the spatial resolution can be as high as the detector spatial resolution (55 µm pixels in our case). To improve the neutron statistics in our analysis we combine the spectra from the neighbouring pixels (typically within ∼1.1 × 1.1 mm area) before the fitting is performed. The reconstructed strain map in this way is similar to an ideal strain map blurred by a running Gaussian filter. It is still possible to observe strain features on the scale of 100 µm, but the absolute values of strain variation are smoothed by that running average. For that reason we only vaguely specify that the spatial resolution of our strain reconstruction is on a sub-mm scale, but it is still more detailed than a discretely pixellated strain map which can be obtained by a fixed-area scanning across the sample. We foresee that the spatial resolution of our technique can be assessed in future investigations by the Fourier ring correlation method, which was applied in a similar way in the assessment of high-resolution neutron imaging (Trtik *et al.*, 2015 ▸).

**Figure 8:**
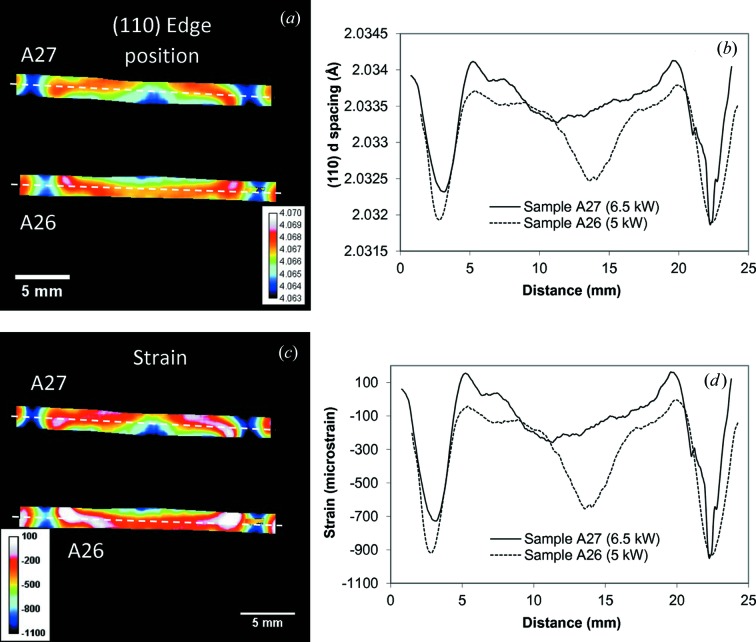
(*a*) The measured distribution of (110) edge position λ within the b.c.c. steel of the Al–steel weld. The map is obtained by fitting an analytical function into the measured spectrum within the ∼1 × 1 mm area around each pixel of the image. The legend shows the colour bar for the Bragg edge wavelengths. (*b*) Cross section (0.5 mm wide) through the edge position map shown by a dashed line in (*a*). The legend indicates the laser power used in welding of each sample. The lattice parameter is calculated as λ/2 according to Bragg’s law. (*c*) Residual strain distribution calculated from (*a*) with the assumption of unstrained lattice value 4.0676 Å. (*d*) Same as (*b*), calculated for the strain.

The distribution of Bragg edge position integrated over the entire sample thickness along the direction of the weld seam is shown in Fig. 8 ▸(*a*) (reconstructed for the steel part of the Al–steel weld). In Figs. 8 ▸(*a*) and 8(*b*) we intentionally did not convert the measured edge position wavelengths into the residual strain values as no specific measurement of unstressed Bragg edge position λ_0_ is available to us at this time. Moreover, the variation of measured Brag edge position λ_0_ can be caused both by residual strain and by the compositional variation in the weld area. These limitations should be taken into account when considering the analysis of residual strain presented in this section. The map of elemental composition can be investigated through the resonance absorption analysis presented in §3.3. This can provide information on macroscopic compositional variation, but with a limited sensitivity, and is limited to certain elements which have resonances at eV to keV energies.

**Figure 9:**
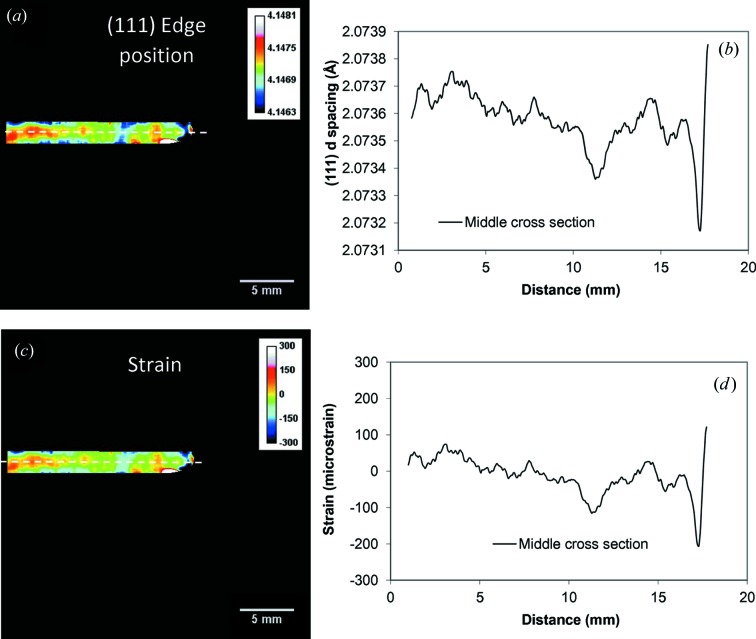
Same as Fig. 8 ▸, except measured for the stainless steel part of the Ti–stainless steel weld. The (111) Bragg edge is used in this analysis.

**Figure 10:**
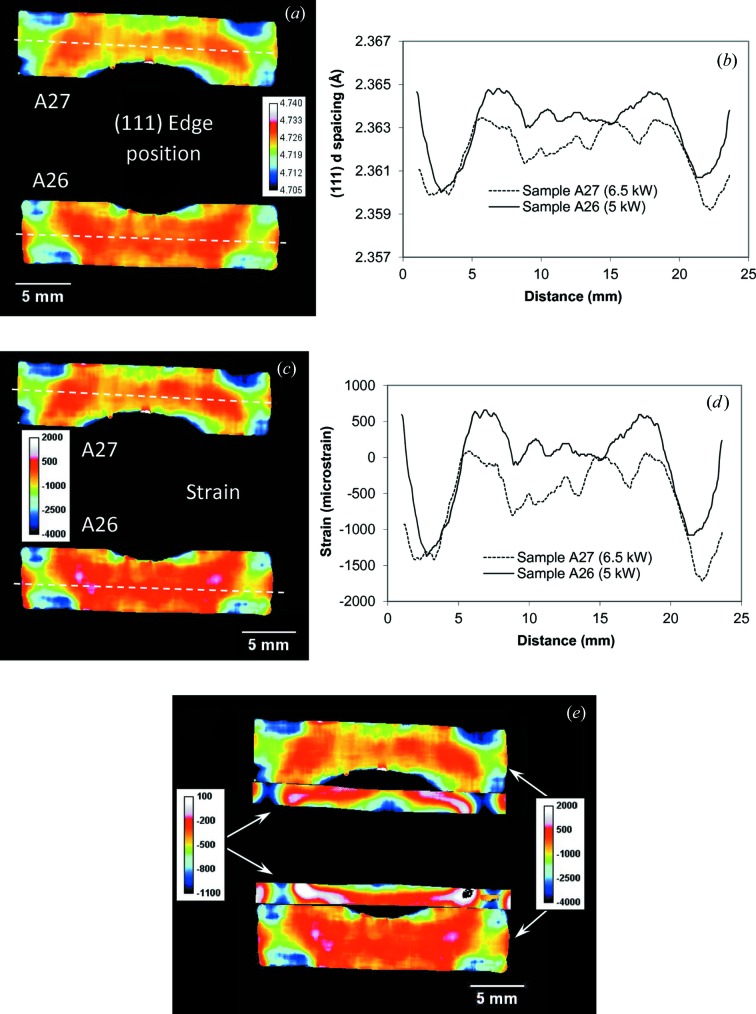
Same as Figs. 8 ▸ and 9 ▸, except measured for the aluminium part of the Al–steel weld. The (111) Bragg edge is used in this analysis. Image (*e*) comprises the combined strain maps. The colours represent different strain values for Al and steel parts of the weld, which are specified by the corresponding colour scale bars shown in the image. This compound strain map is used to demonstrate the spatial correlation between the strains measured in two different parts of the welds. The cross sections across the map of edge positions (*b*) and strains (*e*) are 1 mm wide. No reliable fitting was possible in the heat-affected area of the aluminium part of the weld.

A spatially resolved unstressed lattice parameter *d*_0_ needed for the accurate mapping of residual strain was not properly measured in our experiments. Instead, a reference parent plate value unaffected by the welding heat was taken to analyse the relative residual strain across the welded region. The deficiency of this somewhat arbitrary selection of *d*_0_ value is in the fact that the strain maps shown below are reconstructed with an accuracy of an unknown constant and thus represent only the relative strain variation across the sample. However, the difference in strain values between the various areas should still be accurate and not affected by the shift to an unknown constant. The residual strain values reconstructed with the assump­tion of λ_0_ = 4.0676 Å (measured at the edge of the sample away from the weld area) are shown in Figs. 8 ▸(*c*) and 8 ▸(*d*). The strain distribution maps in Figs. 8 ▸(*c*) and 8 ▸(*d*) depict the relative strain values with respect to the parent plate *d*_0_ and did not take into account the compositional variation which occurred during the welding process. Although these strain maps could not be used to analyse the stress, they provide valuable information that can aid our understanding of the thermal straining of components and structures manufactured using different alloys. The cross section through the strain map shown in Fig. 8 ▸(*d*) shows strong compressive strains at the edges of the molten areas as well as in the very centre of the weld seam for the sample with higher welding power (A27, 6.5 kW power). The lattice parameter and the residual strain distributions within the stainless steel section of the Ti–stainless steel weld are shown in Fig. 9 ▸. Here we assumed that the lattice parameter corresponds to the unstressed value at the very edge of the sample, which was not affected by the welding. No significant strain values were measured except at the very tip of the steel plate close to the welding area. The reconstructed Bragg edge wavelengths and residual strain values for the (111) Al edge are shown in Fig. 10 ▸ for the aluminium part of the weld, where strong compressive strains are also seen at the edges of the heat-affected area. Here it is assumed that the parameter λ_0_ is equal to 4.7265 Å (value measured at the edge of the sample), resulting in strain values varying between ∼1000 and ∼−3000 microstrain. No distinct Bragg edges were measured in the melted Al area, adjacent to the centre of the weld interface between Al and steel. Here the melting and rapid re-solidification of aluminium is expected to produce a strongly textured microstructure, resulting in a complicated transmission spectrum with no obvious Bragg edges. That is why the strain map does not show any values in that region.

**Figure 11:**
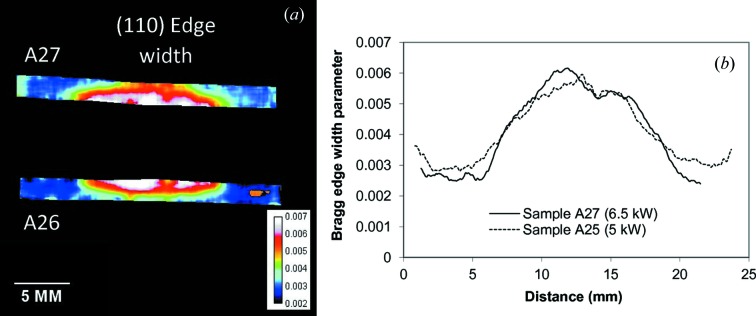
(*a*) The measured distribution of the (110) edge width within the b.c.c. steel of the Al–steel weld. The edge width is obtained by fitting an analytical function into the measured spectra within the 1.1 × 1.1 mm area around each 55 µm pixel of the image. (*b*) A 0.5 mm-wide cross section through the map of edge width.

**Figure 12:**
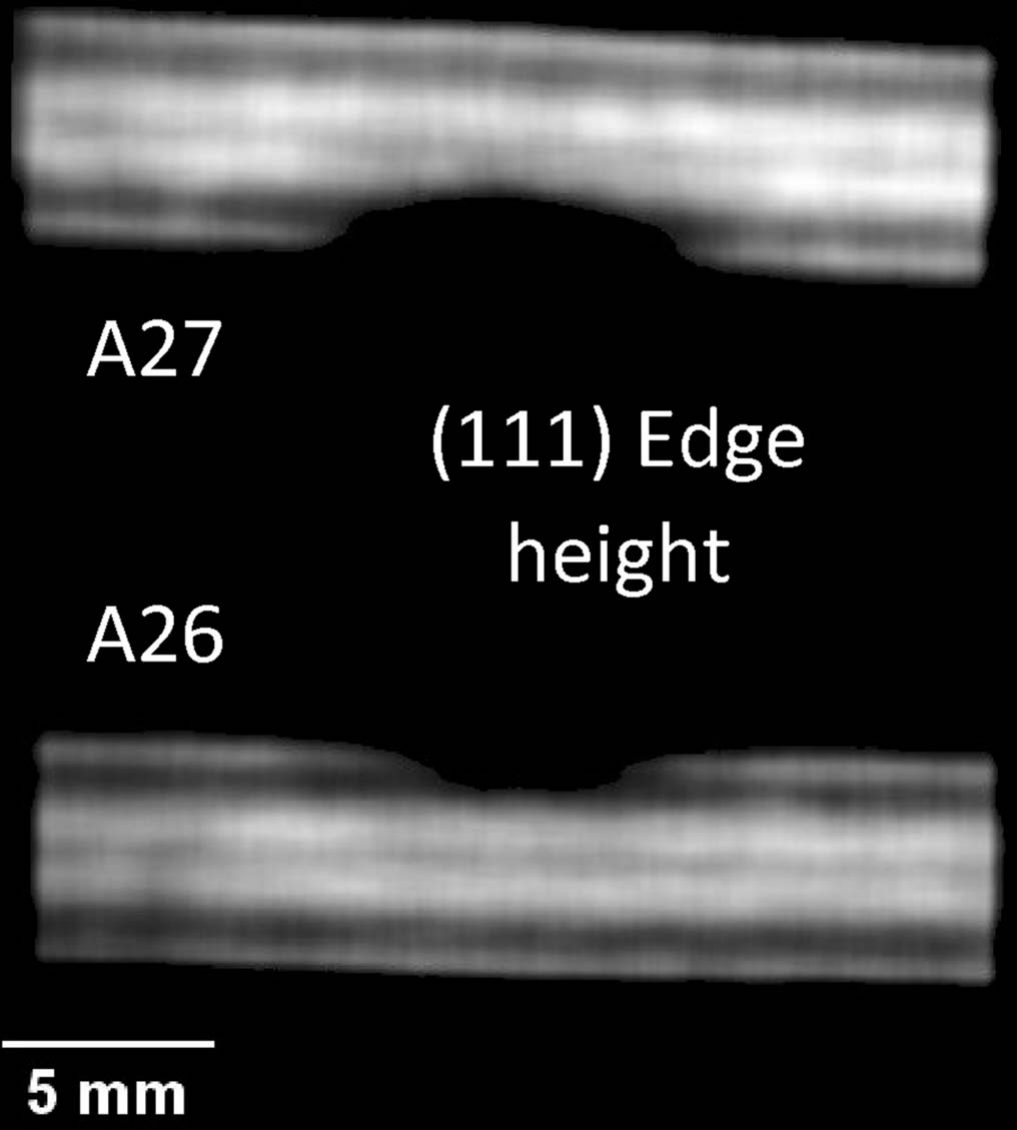
The reconstructed distribution of the (111) edge height parameter of the Al part of the weld, indicating the presence of texture which most likely originated from the rolling step of plate manufacture. The features seen in this image are not seen in the narrow-energy-range images.

The reconstructed map for the width of the Bragg edge for the Al–steel sample is shown in Fig. 11 ▸. That width parameter is correlated to the degree of randomness of grain orientation (or to the presence of preferred orientations). As expected from the images revealing the extent of the heat-affected zone, the width of the Bragg edge increases considerably at the very top of the weld seam, where the re-crystallization process was strongest during the welding and subsequent seam solidification. At the same time, the original texture distribution within the Al plate, caused by the rolling manufacturing process, is seen in the fitted Bragg edge height parameter of Fig. 12 ▸. Here the texture of the original Al plate probably caused by the rolling process during manufacture is clearly visible.

### 3.3. Distribution of elemental composition measured through resonance absorption analysis   

**Figure 13:**
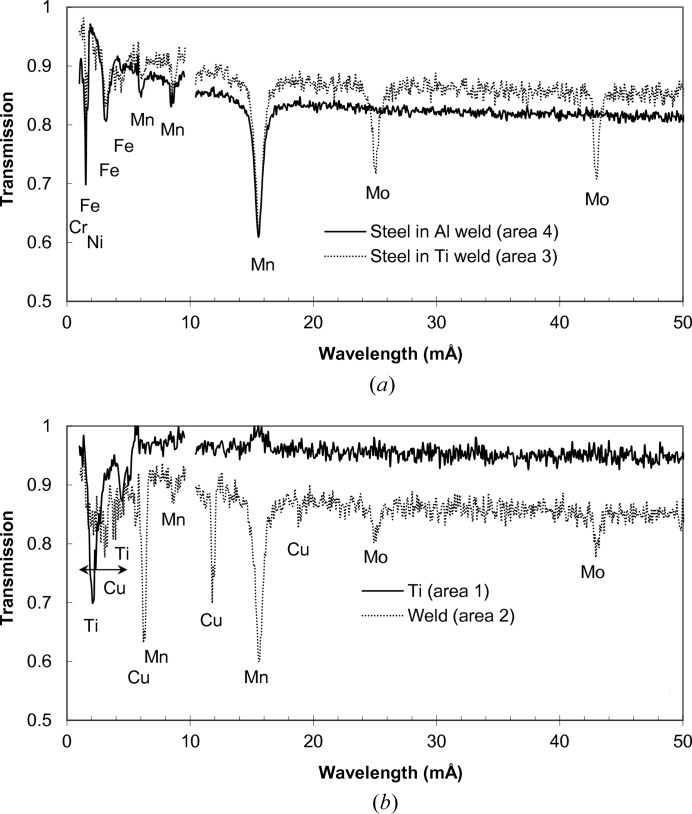
Transmission spectra of weld samples measured in the epithermal rage of energies. The spectra are measured in a ‘face-on’ orientation for the areas shown by the dashed rectangles in Fig. 6 ▸(*a*). Sharp variation of transmission is observed, due to the resonance absorption of neutrons by several elements present in the weld materials. (*a*) Spectra of the stainless steel part of the Ti–stainless steel weld [area 1 in Fig. 6 ▸(*a*)] and the Al–steel weld [area 4 in Fig. 6 ▸(*a*)]. The Mn resonances from both steel and Al parts of the weld are present, as well as Cr resonance from the Al part of the weld. A weak Ni resonance is barely resolved for the stainless steel side of the Ti–steel weld. (*b*) The spectra of Ti [area 3 of Fig. 6 ▸(*a*)] and the weld area of the same sample [area 2 of Fig. 6 ▸(*a*)]. A substantial amount of Mn is present in the weld. The spatial distributions of these elements in the samples are shown in Fig. 14 ▸.

**Figure 14:**
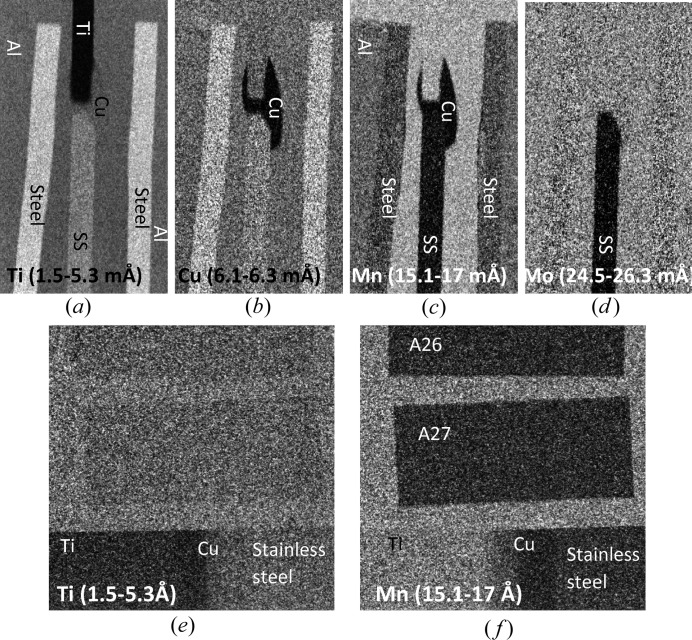
Maps of elemental distribution for the materials which have relatively low resonance absorption energies as seen in Fig. 13 ▸. The range of neutron wavelength at which the images are acquired is indicated in each image. The wide-spectrum images of the same data set are shown in Figs. 4 ▸ and 6 ▸. Images (*a*)–(*d*) are normalized by the image integrated over the [18.7 mÅ, 36.4 mÅ] range of wavelengths, where there are no resonances present. Images (*e*) and (*f*) are normalized by the white-spectrum open-beam image. Even small variations of Mn concentration (*e.g.* 0.5–1.7% in our samples) can be resolved, as seen in image (*c*) (note that the samples have the same 10 mm thickness in that orientation).

Since the entire neutron transmission spectrum was measured in our experiments, including the epithermal range of energies, the mapping of elemental composition within the welds can be performed simultaneously through a resonance absorption analysis. Not all elements have resonance energies that are measurable in our current experimental setups, where the range of measured spectra is limited to wavelengths above 2 × 10^−3^ Å (below ∼20 keV energies) because of the finite width of the initial neutron pulse. The other limiting factors are the presence of background signal in the neutron beam itself and the relatively low detection efficiency of the high-resolution detector in the epithermal range of energies. For some elements (in fact some isotopes of certain elements) exhibiting low-energy (∼eV to ∼keV) resonance absorption our measured spectra can be used to identify their presence in different areas of the sample, and in some cases to quantify their concentrations. Even when only one isotope of a particular element has measurable resonance energies, the presence and/or concentration of that element in each pixel of the image can be obtained as no segregation of isotopes is expected in the weld samples. Fig. 13 ▸ shows the measured transmission spectra for the different areas of our welds specified by the dashed rectangles in Fig. 6 ▸. As seen in these spectra the quantification of iron concentration in these samples is problematic owing to the high resonance energy of iron. However, the resonances of titanium and copper, as well as the additives to steel (Mn, Mo), have substantially large dips in the measured spectra. The narrow-energy images, with corresponding wavelength ranges, shown in Fig. 14 ▸ demonstrate the possibility of performing element-specific imaging through the entire thickness of the sample. Note that neutrons of these high energies have very large penetration capability and thus very thick samples (up to several inches of steel, for example) can be studied nondestructively by this technique. The Ti-specific image of Fig. 14 ▸(*a*) indicates that the diffusion of Ti into the Cu filler area is limited to only a small boundary layer [forming intermetallic compounds seen in the micrograph of Fig. 1 ▸(*c*)] and does not extend far into the weld. Note that, in contrast to other conventional imaging techniques revealing the distribution of elements on the surface, resonance absorption imaging allows investigation of elemental distribution integrated through the entire thickness of the sample. The contrast in the images of Fig. 14 ▸ is due to the resonance absorption by the nuclei and thus it is purely defined by the distribution of the elements, with no sensitivity to their chemical binding or alloying. The boundary of Cu distribution in the weld is seen to be quite sharp, indicating that there was no substantial diffusion of copper into the steel or Ti plates (Fig. 14 ▸ *b*). The presence of Mn in the steel and Al plates in both welds is seen in Fig. 14 ▸(*c*), and the difference between the Mn concentration in different parts of the weld is clearly distinguishable, demonstrating the possibility of quantifying the distribution of that and some other elements to sub-% level for samples of a thickness of a few millimetres to a few centimetres.

## 4. Conclusions   

With this study we demonstrate that energy-resolved neutron transmission imaging can be a very attractive alternative to a number of other conventional techniques, allowing nondestructive investigation of the microstructure and elemental composition and uniformity within the welds. These experiments are made possible thanks to the recent development of bright pulsed neutron sources and high-resolution neutron counting detectors, capable of simultaneous detection of all neutron energies with the time-of-flight method and spanning neutron energies from epithermal to cold energies in one experiment. Simultaneous analysis of diffraction, attenuation and resonance absorption signals measured in each pixel of the acquired data set allows the investigation of microstructure, identification of the presence of defects, and mapping of the elemental composition within relatively thick weld sections with a spatial resolution determined by the pixel size of the detection systems, 55 µm in our current setup. Although these studies can be performed in only a few existing pulsed neutron facilities there will be more dedicated imaging beamlines built in the near future, in particular at the European Spallation Source and the Spallation Neutron Source at Oak Ridge National Laboratory.

## Supplementary Material

A scan through the narrow-band neutron images (between 2.04 and 4.33 Å). Variation of sample transmission with neutron wavelength results in the image contrast which reveals the presence of texture in the copper weld area.. DOI: 10.1107/S1600576716006725/ks5507sup1.avi

## Figures and Tables

**Table 1:** Laser welding parameters used to create the Al–steel samples

Sample number	Laser beam diameter (mm)	Laser power (kW)	Travel speed (m min^−1^)
A26	14.9	5.00	0.40
A27	14.9	6.52	0.40

**Table 2:** CMT parameters used to create the Ti–stainless steel samples with copper welding wire

Sample number	CMT mode	Wire feed speed (m min^−1^)	Travel speed (m min^−1^)
19 CMT	1183	7.0	0.5

**Table:** Aluminium to steel alloy.

	Elements (wt%)											
	Al	Fe	C	Si	Mn	P + S	N	Ti	Cu	Mg	Zn	Cr
XF350 (steel)	0.045	Balance	0.0002	0.015	0.613	0.024	0.017	0.002				
5083-H22 (aluminium)	Balance	0.4		0.4	0.5			0.15	0.1	2.6–3.6	0.2	0.3

**Table:** Titanium to stainless steel alloy.

	Elements (wt% maximum)															
	Si	Fe	Mo	Mn	Ni	Cr	C	P	S	Ti	N	Al	V	O	H	Y
AISI 316 L (stainless steel)	0.45	Balance	2.07	1.73	10	17.2	0.02	0.032	0.01		0.054					
Ti-6Al-4V (titanium)		0.3					0.08			Balance	0.05	6.75	4.5	0.2	0.15	0.05
